# DENTAL WEAR AND TOOTH LOSS IN MORBID OBESE PATIENTS AFTER BARIATRIC SURGERY

**DOI:** 10.1590/0102-672020190001e1458

**Published:** 2019-12-09

**Authors:** Fabiano Duarte AZNAR, Fabio D. AZNAR, José R. LAURIS, Elinton Adami CHAIM, Everton CAZZO, Silvia Helena de Carvalho SALES-PERES

**Affiliations:** 1 Department of Pediatric Dentistry, Orthodontics and Collective Health, Faculty of Dentistry of Bauru, University of São Paulo, Bauru, SP; Brazil; 2 Department of Surgery, Faculty of Medicine, University of Campinas, Campinas, SP, Brazil

**Keywords:** Obesity, Tooth loss, Bariatricsurgery, Tooth wear, Obesidade, Perda de dente, Cirurgia bariátrica, Desgaste dos dentes

## Abstract

**Background:**

Obesity and its surgical treatment have been related with oral diseases**. *Aim*:** To evaluate and compare dental wear and dental loss in eutrophic and morbidly obese patients submitted to Roux-en-Y gastric bypass.

**Method:**

Observational and analytical study with gender and age matching. The sample consisted of 240 patients, divided into four groups: eutrophic (GC=60), morbidly obese (GO=60), operated with up to 24 months (G24=60) and operated on for more than 36 months (G36=60). The following variables were analyzed: race, schooling, economic class, hypertension, diabetes, triglycerides, cholesterol, BMI, weight loss, waist-hip ratio, smoking, alcoholism, tooth loss and tooth wear.

**Results:**

GO presented lower economic class (p=0.012), hypertension (p<0.001), diabetes (p<0.001), cholesterol (p=0.001), BMI (p<0.001), waist-hip ratio (p<0.001) and percentage of weight loss percent (p<0.001) than groups G24 and G36. Dental wear was higher among the II and V sextants.

**Conclusion:**

Individuals submitted to Roux-en-Y gastric bypass, regardless of the surgery period, presented more dental wear on the incisal/occlusal surfaces, and the anterior teeth were the most affected. Dental wear was associated with age and number of missing teeth.

## RESUMO

**Racional:**

Obesidade e seu tratamento cirúrgico têm sido relacionados às alterações bucais.

**Objetivo:**

Avaliar e comparar a ocorrência de desgaste dentário e perda dentária em pacientes eutróficos, obesos mórbidos e submetidos ao bypass gástrico em Y-de-Roux.

**Métodos:**

Estudo tipo observacional e analítico com pareamento de gênero e idade. A amostra foi composta por 240 pacientes, distribuídos em quatro grupos: eutróficos (GC=60), obesos mórbidos (GO=60), operados com até 24 meses (G24=60) e operados com mais de 36 meses (G36=60). Foram analisados raça, escolaridade, classe econômica, hipertensão, diabete melito, triglicerídeos, colesterol, IMC, % de perda de peso, relação cintura-quadril, tabagismo, etilismo, perda dentária e desgaste dentário***.***

**Resultados:**

GO apresentou menor classe econômica (p=0,012), maior proporção de hipertensão (p<0,001), diabete (p<0,001), colesterol (p=0,001), IMC (p<0,001), relação cintura-quadril (p<0,001) e % de perda de peso (p<0,001) que os grupos G24 e G36. O desgaste dentário foi maior entre os sextantes II e V.

**Conclusão:**

Os indivíduos submetidos ao bypass gástrico em Y-de-Roux, independente do período da operação, apresentaram mais desgaste dentário nas superfícies incisais/oclusais, sendo que os dentes anteriores foram os mais afetados. O desgaste dentário esteve associado à idade e ao número de dentes perdidos.

## INTRODUCTION

Obesity is understood as a process of accumulation of excessive fat in the body that results in degradation of homeostasis and causes biochemical and physiological dysfunctions of tissues. Surgical treatment is the most effective way of long-term weight loss^3^. Different surgical techniques have been performed to treat it. However, Roux-en-Y gastric bypass (BGYR) has been considered a gold standard. With the reduction of the size of the stomach to a small pouch connected to the small intestine, excluding the duodenum and portion of the jejunum, it induces restrictive and desabsortive effects that lead to weight loss^19^. The long term results of BGYR are well documented, both in terms of weight loss, improvement or resolution of obesity-related comorbidities, risk factors and improved quality of life^6^.

There is growing interest in the relationship between BMI and oral status, since both are important public health concerns^21^. Bariatric surgery has been related to improvements in systemic conditions and worsening of oral conditions, specifically increased gingivitis^24^ and periodontitis^23^, tooth wear^15^ and caries lesions^17^.

The increase in the life expectancy of the population coupled with the decline in the prevalence of dental caries contributed to the maintenance of a greater number of dental elements present in adults and the elderlies. More dental surfaces are exposed, and may suffer wear and tear^10^.

Dental wear is the gradual loss of substance from the dental element, without the involvement of the carious process, without interference from the action of microorganisms or trauma. Changes in lifestyle, diet and behavior, play a key role in tooth wear. Dental wear is the loss of hard dental tissue due to erosion, attrition, abrasion and abduction processes^17^. The dental surface can suffer wear as a result of the natural process or triggered by changes, which the teeth are exposed. This process is multifactorial, involving chemical and mechanical factors, in a continuous and gradual manner^22^.

The presence of teeth has been used as an indicator of oral health, and maintaining 20 permanent teeth as a function protects functional, aesthetic and phonetic conditions throughout life^27^. Individuals who do not have this condition may suffer from masticatory problems, food restriction and inadequate nutrient intake^5^. However, edentulism is the complete loss of teeth, occurring in one or both dental arches, partial or total, respectively. Tooth loss is one of the main oral problems and due to its high prevalence, the aesthetic, functional, psychological and social damages that it causes to the individual^26^.

The oral conditions of morbidly obese individuals, both pre and postoperative, are not clearly evidenced in the scientific literature, since these individuals may have alterations related to metabolism, psychosocial and environmental factors. Few studies have related dental wear and tooth loss comparing the individual in the obese phase and after the operation of obesity.

Thus, the present study aimed to evaluate the occurrence of dental wear and tooth loss in eutrophic, obese individuals before and after bariatric surgery.

## METHODS

The present study was a cross-sectional and analytical observational study, developed in the period from April/2015 to April/2017, during the period in which eutrophic, pre- and post-surgical morbidly obese patients were enrolled up to 24 months and more than 36 months of BGYR, by SUS at the Clinical Hospital of the State University of Campinas, Campinas, SP, Brazil. The project was approved by the Committee of Ethics in Research with Human Beings of the Clinical Hospital of the Institution (Ethics Committee number: 73245517.0.0000.5404).

The sample size was calculated based on the power of the test of 80% and the coefficient of confidence of 95%. The sample consisted of 240 patients (eutrophic=60, obese=60, <24 months operated=60, >36 months operated=60), respecting the rule that the number of cases of the smallest binary logistic regression by the number of independent variables resulted in an amount of not less than 20^7^.

The sample consisted of 60 patients divided into four groups: GO - morbid obese; G24 - morbid obese with 24 months of surgery; G36 - with 36 months of surgery; GC - eutrophic individuals. The groups were matched according to gender and age.

The inclusion criteria were for GO BMI greater than or equal to 40 kg/m^2^ and for GC or BMI between 18.5-24.99 kg/m^2^. The G24 and G36 groups underwent bariatric surgery using the gastric by-pass technique, in addition to having undergone only one surgery to treat obesity.

Exclusion criteria were pregnancy, cancer, surgical complications and/or immunosuppressive drugs.

The calibration training of the examiner for the tooth wear index-IDD^22^ was performed before starting the clinical exams. The calibration training was conducted by a standard examiner, experienced in epidemiological surveys and the activities were divided into theoretical and practical, involving training and calibration exercises, comprising a total of six periods. The first periods were used to explain the codes, criteria and behaviors adopted for the study. After this period, the practice of exercises was initiated through visual exposure of clinical cases by the standard examiner; then the evaluations and possible discussions were carried out. After this practical exercise, a clinical demonstration was performed on how the exams should be done and the calibration itself, with 10 volunteers selected at the Faculty of Dentistry of Bauru, SP, Brazil. After data collection, a general discussion was conducted to make sure that the examiner became familiar with the procedures^21^. For Kappa calculation, 10% of the patients were reassessed on another day. The entire sample was evaluated by a single examiner, with Kappa found intra-examiner >0.96, demonstrating excellent agreement^9^.

The nutritional assessment of the individuals was performed by measuring the anthropometric measures weight and height, according to the techniques recommended by the WHO^29^, and from these values calculated the BMI. For the classification of nutritional status, the cut-off points defined by the WHO were used^29^, that is: from 18.5 to 24.9 kg/m² normal (eutrophic); 25 to 29.9 kg/m² overweight; 30 to 34.9 kg/m² obesity grade I; 35 to 39.9 kg/m² obesity grade II and over 40 kg/m² obesity grade III (morbid obesity).

Waist circumferences (CC) and hip circumference (CQ) were obtained using a tape in centimeters, measured with the individual wearing thin clothing, relaxed abdomen, in an orthostatic position, with the feet together and arms at the side of the body. To measure the circumference of the hip, the level of the point of greatest circumference of the gluteal region and for the measurement of the waist, the midpoint between the iliac crest and the last costal arch, was measured. To calculate the waist and hip ratio (WHR), the waist circumference was divided by the hip circumference. The waist circumference was divided by the circumference of the hip. It was considered a high risk to health in the man WHR above 0.95; moderate risk between 0.90 and 0.95; low, less than 0.90. In women the high risk would be values greater than 0.85; moderate risk between 0.80 and 0.85; low, less than 0.80^29^.

All the surgical procedures were performed by the same medical team and adopting the same technique, which was the BGYR. The sociodemographic characteristics, comorbidities and life habits, anthropometric evaluation were collected and recorded in a specific file.

The patients were submitted to dental examination for the identification of tooth wear and tooth loss with the aid of intra-oral mirror and wooden spatula, previously sterilized, visual examination without obtaining images.

For dental wear, the index adjusted by Sales Peres^22^ was used, which allows the evaluation of the prevalence and severity of wear. All dental surfaces (buccal, palatal/lingual, incisal/occlusal) of all teeth were examined, using “0” scores for a surface that shows no wear; “1” wear with enamel-only wrapping; “2” wear with dentine exposure; “3” secondary dentin or pulpal exposure; “4” dental restoration due to wear and “9” score for the surface that could not be evaluated for having extensive cavities or restorations or great loss of structure. Dental wear was measured according to the sextant involved and its totality per individual. The examination to identify dental wear was preceded by drying of dental surfaces, which was performed with a transportable device with two triple syringes, facilitating visual diagnosis. Ambient light was used and complemented by the help of artificial light.

The presence or absence of teeth was recorded to identify regions and arches where these absences were concentrated. They were grouped in total of lost teeth, by arcade and by group of teeth (incisors, canines, premolars and molars).

### Statistical analysis

Statistical tests were used according to the variables (BMI, age, weight loss, waist/hip ratio and missing teeth), parametric tests and qualitative variables (gender, race, schooling, economic class, hypertension, diabetes type 2, triglycerides, cholesterol, alcoholism and smoking) nonparametric. The outcome variables were dental wear and tooth loss. The variables of exposure were gender, race, schooling, economic class, hypertension, type 2 diabetes, triglycerides, cholesterol, alcoholism, smoking, BMI, age, % weight loss and waist/hip ratio. Descriptive statistical analysis was applied to obtain the absolute and relative frequencies. Bivariate analysis was performed using the Chi-Square, Mann-Whitney, ANOVA, Tukey and Kruskal-Wallis tests. To verify the association between obesity and oral outcomes (dental wear and tooth loss), the multivariate linear regression model was used, calculating the odds ratio and the 95% confidence interval. Age, gender, BMI, % of weight loss, cervical circumference and waist-to-hip ratio were included as independent covariates in the multiple linear regression analysis, when they obtained p<0.20. Analyzes were conducted using the Statistica 25.0 program for Windows, adopting a significance level of 5% (p<0.05) for all tests.

## RESULTS

Although there were more women in the sample, there were no statistically significant differences among the groups for ethnicity (p=0.135), schooling (p=0.108), triglycerides (p=0.078), smoking (p=0.568) and alcoholism (p=0.712). However, GO presented lower economic class (p=0.012) than the other groups. In addition, this group also showed a higher percentage of hypertension (p<0.001), diabetes (p<0.001) and cholesterol (p=0.001, Table 1).

**TABLE 1:** Sociodemographic characteristics, comorbidities and life habits among obese individuals (GO), operated up to 24 months (G24), operated after 36 months (G36) and eutrophic (CG).

VARIABLE	CHARACTERISTICS	GO (n=60)	G24 (n=60)	G36 (n=60)	GC (n=60)	TOTAL (n=240)	p
Gender	Female	48 (80%)	48 (80%)	48 (80%)	48 (80%)	192 (80%)	0.682^¥^
	Male	12 (20%)	12 (20%)	12 (20%)	12 (20%)	48 (20%)	
Race	Branca	33 (55%)	43 (71,7%)	42 (70%)	39 (65%)	157 (65,4%)	0.135^¥^
	Negra	12 (20%)	9 (15%)	8 (13.3%)	4 (6.7%)	33 (13.8%)	
	Parda	15 (25%)	8 (13.3%)	10 (16.7%)	17 (28.3%)	50 (20.8%)	
Education	Elementary school incomplet	15 (25%)	9 (15%)	7 (11.7%)	8 (13.3%)	39 (16.3%)	0.108^£^
	Elementary school complet	2 (3.3%)	5 (8.3%)	9 (15%)	7 (11.7%)	23 (9.6%)	
	Hight school incomplet	7 (11.7%)	1 (1.7%)	4 (6.7%)	3 (5%)	15 (6.3%)	
	Hight school complet	30 (50%)	32 (53.3%)	27 (45%)	24 (40%)	113 (47.1%)	
	Higher education incomplet	2 (3.3%)	4 (6.7%)	4 (6.7%)	5 (21.7%)	15 (6.3%)	
	Higher education complet	4 (6.7%)	9 (15%)	9 (15%)	13 (21.7%)	35 (14.6%)	
Financial Class	Until 1 salary	15 (25%)	9 (15%)	11 (18.3%)	11 (18.3%)	46 (19.2%)	0.012^£^
	From1 to 2 salaries	34 (56.7%)	23 (38.3%)	27 (45%)	22 (36.7%)	106 (44.2%)	
	From 2 to 3 salaries	7 (11.7%)	14 (23.3%)	11 (18,3%)	11 (18.3%)	43 (17.9%)	
	From 3 to 4 salaries	2 (3.3%)	8 (13.3%)	5 (21.7%)	9 (15%)	24 (10%)	
	From 4 to 5 salaries	0 (0%)	2 (3.3%)	3 (5%)	3 (5%)	8 (3.3%)	
	Above 5 salaries	2 (3.3%)	4 (6.7%)	3 (5%)	4 (6.7%)	13 (5.4%)	
Hypertension	No	28 (46.7%)	51 (85%)	54 (90%)	55 (91.7%)	188 (78.3%)	<0.001^¥^
	With	32^a^ (53.3%)	9^b^ (15%)	6^b^ (10%)	5^b^ (8,3%)	52 (21.7%)	
Diabetes	No	45 (75%)	57 (95%)	59 (98.3%)	60 (100%)	221 (92.1%)	<0.001^¥^
	With	15^a^ (25%)	3^b^ (5%)	1^b^ (1.7%)	0^b^ (0%)	19 (7.9%)	
Triglycerides	No	55 (91.7%)	57 (95%)	59 (98.3%)	60 (100%)	231 (96.3%)	0.078^¥^
	With	5 (8.3%)	3 (5%)	1 (1.7%)	0 (0%)	9 (3.8%)	
Cholesterol	No	48 (80%)	55 (91.7%)	60 (100%)	56 (93.3%)	219 (91.3%)	0.001^¥^
	With	12^a^ (20%)	5^a^ (8.3%)	0^b^ (0%)	4^ac^ (6.7%)	21 (8.8%)	
Smoking	No	49 (81.7%)	54 (90%)	53 (88,3%)	52 (86.7%)	208 (86.7%)	0.568^¥^
	With	11 (18.3%)	6 (10%)	7 (11.7%)	8 (13.3%)	32 (13.3%)	
Alcoholism	No	52 (86.7%)	52 (86.7%)	48 (80%)	51 (85%)	203 (84.6%)	0.712^¥^
	With	8 (13.3%)	8 (13.3%)	12 (20%)	9 (15%)	37 (15.4%)	

Sextants withsame letter (a, b), do nothavesignificant statistical difference each other; ¥=Chi-Square test and proportions; £=Mann-Wihitney test

There were significant differences between groups in relation to BMI (p<0.001) and between % weight loss between G24 and G36 (p<0.001). GO showed higher values in the waist/hip ratio than the other groups (p<0.001, Table 2).

**TABLE 2:** Comparison of age, BMI, weight loss and waist-to-hip ratio between obese (GO), operated up to 24 months (G24), operated after 36 months (G36) and eutrophic (CG)

VARIABLE	GO (n=60) $\bar{x}\bar{x}$dp	G24 (n=60) $\bar{x}\bar{x}$dp	G36 (n=60) $\bar{x}\bar{x}$dp	GC (n=60) $\bar{x}\bar{x}$dp	p
Age	38.083^a^ ± 8.158	37.900^a^ ± 7.852	38.583^a^ ± 7.308	36.700^a^ ± 8.110	0.604^§^
BMI	49.604^a^ ± 7.774	30.021^b^ ± 5.116	29.034^b^ ± 6.743	22.214^c^ ± 1.951	<0.001^§^
% Lossweight		40,552^a^ ± 10.703	33.162^b^ ± 12.000		<0.001^§^*
WHR	0.910^a^ ± .095	0,881^b^ ± 0.110	0.844^b^ ± 0.094	0.817^b^ ± 0.083	<0.001^§^

Sextants with same letter (a, b), do not havesignificant statistical difference each other; §=ANOVA and Tukey test; *=groups with weight loss measured post surgical

The number of teeth lost according to the groups were: GO=3.70±4.68, G24=2.78±4.14, G36=3.70±6.44 and GC=3.03±5.07. ANOVA followed by the Tukey test showed no significant differences between groups for lower loss teeth (p=0.4324) and higher (p=0.3886).

The total tooth wear index showed that 2868 (65.58%) of the faces showed no wear, and one third of the total faces evaluated had wear (34.42%), 1448 (33.11%) presented wear on occlusal/incisal surfaces and 57 (1.31%) wear on the buccal/lingual surfaces. There were no significant differences between groups in relation to tooth wear by both sextants and total (p=0.448). However, there was greater wear among the sextants II and V corresponding to the upper and lower anterior teeth (Table 3).

**TABLE 3:** Comparison of total dental wear and sextants among obese individuals (GO), operated up to 24 months (G24), operated after 36 months (G36) and eutrophic (CG)

PLACE	GO n $\bar{x}\bar{x}$dp	G24 n $\bar{x}\bar{x}$dp	G36 n $\bar{x}\bar{x}$dp	GC n $\bar{x}\bar{x}$ dp	Total n $\bar{x}\bar{x}$dp	p entre grupos
Sextant I	59 0.411^a^ ± 0.125	59 0.412^ab^ ± 0.121	56 0.425^a^ ± 0.119	58 0.378^a^ ± 0.077	232 0.406^a^ ± 0.113	0.145^§^
Sextant II	59 0.599^b^± 0.125	59 0.564^c^ ± 0.200	56 0.641^b^ ± 0.178	57 0.609^c^ ± 0.143	231 0.603^c^ ± 0.177	0.133^§^
Sextant III	59 0.415^a^ ± 0.125	59 0.385^b^± 0.095	52 0.411^a^± 0.127	580.381^a^± 0.083	228 0.398^a^ ± 0.122	0.316^§^
Sextant IV	59 0.444^a^± 0.125	57 0.460^a^± 0.150	55 0.449^a^ ± 0.129	59 0.448^b^ ± 0.160	230 0.450^b^ ± 0.161	0.958^§^
Sextant V	59 0.615^b^± 0.125	59 0.596^c^± 0.160	58 0.602^b^ ± 0.104	59 0.626^c^± 0.079	235 0.609^c^± 0.118	0.508^§^
Sextant VI	59 0.433^a^ ± 0.125	59 0.455^a^± 0.149	57 0.457^a^± 0.154	58 0.420^ab^± 0.132	233 0.441^b^ ± 0.146	0.455^§^
Total	59 0.510 ± 0.105	59 0.498 ± 0.107	58 0.526 ± 0.099	59 0.505 ± 0.072	235 0.510 ± 0.097	0.448^§^

Sextants with same letter (a, b), do not havesignificant statistical difference each other; ANOVA and Tukey test

There were statistically significant differences between almost all sextants (p<0.001) when compared to total wear. Only the relation between the sextants I and III (p=0,995), II and V (p=0,953) and IV and VI (p=0,979) did not obtain statistically significant differences (Table 4).

**TABELA 4:** Comparison of the total dental wear of the groups among the sextants.

SEXTANTS	I	II	III	IV	V	VI
I	x	< 0.001	0.995	< 0.001	< 0.001	0.007
II	< 0.001	x	< 0.001	< 0.001	0.953	< 0.001
III	0.995	< 0.001	x	< 0.001	< 0.001	< 0.001
IV	< 0.001	< 0.001	< 0.001	x	< 0.001	0.979
V	< 0.001	0.953	< 0.001	< 0.001	x	< 0.001
VI	0.007	< 0.001	< 0.001	0.979	< 0.001	x

ANOVA and Tukey test

In the multiple linear regression analysis of tooth wear, there were statistically significant differences between tooth wear for age (p=0.000) and tooth loss (p=0.001, Table 5). 

**TABLE 5:** Multiple linear regression analysis for dental wear with the other variables

Model	Coeficients not adjusted		Adjusted coeficients	t	p	Confidence intervalof B (95%)	
	B	Error	Beta			Minimum	Maximum
(Constant)	0.282	0.062		4.519	0.000	0.159	0.404
GO	-0.010	0.032	-0.046	-0.318	0.751	-0.073	0.053
G24	-0.009	0.017	-0.043	-0.548	0.585	-0.043	0.025
G36	0.013	0.017	0.059	0.781	0.435	-0.020	0.047
Age	0.005	0.001	0.427	6.566	0.000	0.004	0.007
Sex	0.024	0.017	0.094	1.450	0.149	-0.009	0.057
Black	-0.020	0.016	-0.073	-1.234	0.218	-0.053	0.012
Brown	0.003	0.014	0.011	0.189	0.850	-0.026	0.031
Education	0.001	0.004	0.011	0.170	0.865	-0.007	0.009
Financial Class	0.005	0.005	0.072	1.127	0.261	-0.004	0.015
Hypertension	0.002	0.016	0.009	0.123	0.902	-0.030	0.034
Diabetes	0.019	0.025	0.052	0.774	0.440	-0.030	0.069
Triglycerides	-0.041	0.034	-0.081	-1.202	0.231	-0.108	0.026
Cholesterol	-0.006	0.023	-0.019	-0.272	0.786	-0.052	0.039
Smoking	0.004	0.017	0.016	0.268	0.789	-0.028	0.037
Alcoholism	-0.003	0.016	-0.013	-0.217	0.828	-0.034	0.028
BMI	0.000	0.001	0.047	0.375	0.708	-0.002	0.002
WHR	-0.019	0.069	-0.020	-0.280	0.780	-0.155	0.116
Loss teeth	0.006	0.002	0.220	3.327	0.001	0.002	0.009

In the analysis of effect of the groups, the control group was the reference; in the analysis of race effect the white was the reference

## DISCUSSION

The results of the present study showed that individuals submitted to BGYR present more dental wear on the incisal/occlusal surfaces. In the studied sample, regardless of the time of operation, state of normality or obesity, it was possible to observe greater dental wear in anterior teeth, a fact already identified in the literature^26^. Tooth loss did not vary among the groups studied.

In this study, there was greater participation of women, white race, incomplete average schooling and economy class up to two minimum wages, without significant differences between groups. These findings reinforce that presented in other studies, in which about 80% of the population who undergo bariatric surgery are women^16^. The justification for this fact is based on the greater collection of society in relation to aesthetic standards for women in association with concern to improve the quality of life. However, it must be evidenced that the prevalence of obesity in Brazil is similar between genders^2^.

In the present study, significant differences were identified for anthropometric evaluation between the GO and GC groups; between groups G24 and G36 there were no significant differences. It is noteworthy that the patients submitted to bariatric surgery did not present differences between 24 months or more than 36 months of operated. Afterwards, some behaviors began to be adopted, which directly interfered in the loss of weight and caused significant impact in the operated patients. Both the development of positive behavioral changes occur, including the cessation of negative behaviors or the increase of positive behaviors, which directly affect the amount of weight loss. However, you can change several of these behaviors, such as starting self weighting and stopping eating when you are satisfied or eating continuously during the day as an additive. Regarding the literature, it was shown that after bariatric surgery, there is a reduction in the intake of calories^8^ and fat^13^, better dietary adherence^4^, reducing the intake of snacks, decreasing food cravings. These factors seem to justify the findings of the present study in the groups after bariatric surgery, since the other conditions and habits did not present significant differences between the studied groups.

Among dental conditions, tooth loss is far from being solved in the Brazilian population. The Brazilian epidemiological survey^1^ for the age group of 35-44 years showed that between 2003 and 2010 there was reduction of lost teeth in adults decreased from 13.5 to 7.4, showing that 7.3% of adults had missing dental elements. The need for a prosthesis in the population in this age group was 68.8%^1^. In this study, it was observed that there was a mean loss of 3.3 teeth in adults, which is lower than that found in the last Brazilian epidemiological survey. Among the groups studied, the eutrophic group had the lowest percentage of tooth loss and the obese had the lowest social status^30^.

One of the justifications for the reduction of tooth loss observed in adults is possibly the combination of improved socioeconomic conditions, especially education, and the health system such as exposure to fluoridation and fluoridation of fluoride dentifrices. Poorer and less educated individuals live in locations with lower water fluoridation coverage and dental services, brush their teeth less frequently^19^, consume more sugar, animal foods, saturated fat, sugar, and have less time for physical activity^11^. All these factors contribute to an increase in the prevalence and progression of dental caries and periodontal disease and, consequently, the tooth loss resulting there from. In this way, the most vulnerable populations must have priority attention, alongside universal measures^19^.

The absence of teeth can trigger some degree of temporomandibular dysfunction and that oral rehabilitation is able to act positively in reducing the severity of these disorders in these patients^21^.

Several factors may influence the occurrence of dental wear, such as gastroesophageal reflux, bruxism, salivary pH and age^18^. In the present study, there was a higher prevalence of wear on the anterior teeth and the incisal/occlusal surface, which indicates the occurrence of attrition. This phenomenon can be caused by the contact surfaces involving enamel and dentin, being a result of the adaptive process that can be explained by the high presence of parafunctional habits, such as bruxism, in which excessive vertical forces of long duration and horizontal frictional forces occur^14^. Bruxism has been reported as a physical manifestation of stress and anxiety^28^ and this, in turn, is closely related to obesity. There are two distinct circadian phenotypes for bruxism: sleep bruxism and bruxism, which are considered separate entities due to the supposed difference in their cause and phenotypic variance. The recently proposed causes of bruxism appear to be a combination of genetic and environmental factors, with epigenetics providing a robust framework for investigating these interactions.

Regarding the wear surface, in this study enamel wear was more prevalent in GC (eutrophic) and in dentin in G24 and G36 (24M and 36M after the operation). These results resemble those of Yamashita^30^ in which the authors found greater dentin wear in the obese and higher enamel in the eutrophic.

Erosive wear occurs in the occlusal and smooth surfaces, considering that this lesion arises due to chemical demineralization with the participation of physical factors, such as friction and often stress. Recent review has shown that only in patients with gastroesophageal reflux disease and eating disorders associated with vomiting can a clear impact on the prevalence of erosion be found^25^. Allied to this fact, people who consume acidic foods and drinks, have a greater risk of erosion. Those with reflux disease are more likely to develop tooth erosion and halitosis^12^.

It should be noted that erosion wear can promote widespread wear and be related to other types of wear. Many authors have considered abrasion as a natural phenomenon, which could become excessive when it is associated with friction and erosion^14^.

The linear regression for dental wear showed that there was a significant association between age and tooth loss in the groups studied. This reinforces the scientific evidence that wear and tear worsens with age and can be one more factor for tooth loss. On the other hand, the loss of some teeth can overwhelm the teeth present, especially in cases of bruxism, causing increased wear or its progression. Future studies should be conducted to understand the route of occurrence of this outcome in obese patients before and after bariatric surgery.

Some limitations in the present study merit consideration. Initially, because it is a cross-sectional study, not allow a causal relationship to be made. Another point is the fact that it is a convenience sample restricted to the study site, making it difficult to generalize the findings. As wear accumulates over a long period, longitudinal studies could provide more conclusive evidence about the role and interaction of different risk factors for the development and/or progression of wear. To further understand the progression of tooth wear, more long-term clinical studies are needed and the effects of long-lasting acidic diets or excessive consumption of acidic foods over a period of time are considered. Future follow-up research should focus your research on behavioral habits, hygiene, and consumption of acidic products.

As strengths, the clinical implications for obese and bariatric patients are emphasized, which are based on dental follow-up from the preoperative period, to provide better recovery and mastication adaptation throughout the postoperative period, to contribute to the treatment and prevention of injuries of the oral cavity. 

## CONCLUSION

Dental wear was more prevalent among patients submitted to BGYR, regardless of the postoperative period, and the incisal surfaces of the upper and lower anterior teeth were the most affected. The tooth loss did not show significant differences between the groups. Dental wear was associated with age and number of missing teeth. 
